# Physical Activity Improves Anxiety and Apathy in Early Parkinson's Disease: A Longitudinal Follow-Up Study

**DOI:** 10.3389/fneur.2020.625897

**Published:** 2021-01-15

**Authors:** Samuel Yong-Ern Ng, Nicole Shuang-Yu Chia, Mirza Masoom Abbas, Ehsan Seyed Saffari, Xinyi Choi, Dede Liana Heng, Zheyu Xu, Kay-Yaw Tay, Wing-Lok Au, Eng-King Tan, Louis Chew-Seng Tan

**Affiliations:** ^1^Department of Neurology, National Neuroscience Institute, Singapore, Singapore; ^2^Parkinson's Disease and Movement Disorders Centre, NPF International Center of Excellence, National Neuroscience Institute, Singapore, Singapore; ^3^Centre for Quantitative Medicine, Duke-NUS Medical School, Singapore, Singapore

**Keywords:** physical activity, non-motor, anxiety, apathy, early Parkinson's disease

## Abstract

**Objective:** In a prospective study, we investigated the association between physical activity and various motor, non-motor outcomes, and quality of life in early Parkinson's disease (PD) participants in the PD Longitudinal Singapore Study.

**Background:** Prospective studies that examined the association between physical activity and motor and non-motor domains in early PD are lacking.

**Methods:** 121 PD participants were followed-up prospectively to evaluate the association of physical activity with various symptom domains. The Physical Activity Scale for the Elderly (PASE) was used to measure physical activity annually. PD-related symptoms were categorized by motor, non-motor, and quality of life measures. Multivariate regression with gain score analysis was performed to understand the association of baseline PASE scores with the change of each variable at 1-year follow-up.

**Results:** Higher baseline PASE scores (greater activity) were associated with a younger age, lower MDS-UPDRS motor scores, a smaller levodopa equivalent daily dose, better attention and memory scores, and better QoL. Activity scores in early PD declined on follow-up. Multivariate analysis revealed higher baseline physical activity to be associated with decreased anxiety and apathy scores at 1-year follow-up, after adjusting for demographic variables and medications.

**Conclusion:** We demonstrated that higher baseline physical activity was associated with improved anxiety and apathy symptoms in early PD over a 1-year period.

## Introduction

Parkinson's disease (PD) is a debilitating neurodegenerative disorder characterized by bradykinesia, rigidity, tremor, and postural instability. Some patients also have numerous non-motor symptoms, affecting their overall quality of life. Physical activity may be beneficial in the elderly population, including those with PD (1, 2). Some have suggested that increased physical activity can reduce the risk of developing PD and slow disease progression (3, 4). However, physical activity in PD is often limited due to various social, co-morbid and disease related factors (5), and disease progression will further limit their activity. In patients with moderate PD, low physical activity was associated with increased disease severity, gait imbalance and impairment in activities of daily living (5, 6).

While various exercise regimens have been proposed to be useful in PD and in general population settings (7–12), most of these studies had not examined patients longitudinally, did not investigate non-motor symptoms (10), did not take into account overall physical activity (7); or did not have baseline physical activity scores for comparison (12). To address these gaps in knowledge, we conducted a longitudinal study to investigate the relationship between baseline physical activity and multiple domains: motor, non-motor, and quality of life in early PD patients over a period of 1 year. Specifically, we sought to understand the role of physical activity on PD non-motor features in early disease.

## Methods

### Study Design, Setting, and Participants

A total of 121 PD were recruited, as part of the Parkinson's Disease Longitudinal Singapore (PALS) Study. The PALS Study is a prospective longitudinal cohort study that has been described in detail previously (13, 14). Briefly, MDS-UPDRS Part 3 (motor score) are scored thrice yearly, while non-motor, quality of life, and physical activity assessments are comprehensively assessed annually. In the present study, the baseline and follow-up data of the cohort were used for analysis.

PD participants were defined as having early PD with (1) having cardinal motor symptoms ≤ 2 years and (2) having a diagnosis of ≤ 1 year using the National Institute of Neurological Disorders and Stroke (NINDS) criteria, and were recruited from the movement disorder specialist clinics at the National Neuroscience Institute of Singapore from 2014 to 2016. Participants had to have more than 6 years of education and were able to read and write English or Mandarin to enroll in the study. Exclusion criteria from participation included presence of major medical conditions that prevented regular longitudinal follow-up or orthopedic abnormalities that could affect study outcomes. The ethical committee approval was provided by the Singapore Health Services Centralised Institutional Review Board (CIRB Ref: 2019/2433) and a written informed consent was taken from participants before enrolling in the study.

### Data Collection

The demographic variables (including age, gender, ethnicity, marital status, and years of education) were noted during the baseline visit of the study. During each assessment, the presence of co-morbidities and medication (including LEDD: Levodopa Equivalent Daily Dose) use were noted.

#### Physical Activity Scale for the Elderly

The Physical Activity Scale for the Elderly (PASE) is a brief and widely-used scale for measuring physical activity in elderly (15). It has been corroborated with various objective measures of physical activity. The PASE consists of 26 self-reported questions addressing the intensity and frequency of leisure, household and occupational activities over the past week, with various but non-exhaustive examples given for each category. The test-retest reliability coefficient of the PASE (0.75) exceeds most reported scales for physical activity. The PASE uses empirically weighted scoring across the various activities giving higher scores for more intense forms and duration of activities, in continuous scale with scores ranging from 0 to 793. A higher score is suggestive of greater physical activity (15). It has been validated in normal adults (mean score: 102.9 + 64.1 in >65 years) and people with different ethnicities, including the Chinese (16). PASE has also been validated in neurological disorders like stroke and used in previous PD studies (17, 18). The PASE was used in our cohort as an independent variable that is an effective measure of baseline daily physical activity.

#### Other Motor and Non-motor Dependent Measures

All PALS participants had global cognition was assessed via the Montreal Cognitive Assessment (MOCA). The MOCA was chosen as a highly regarded and sensitive instrument that can pick out cognitive impairment and in particular, cognitive changes in PD (19).

The non-motor and mood measures collected included the Hospital Anxiety and Depression Scale (HADS), Apathy Scale (AS), Fatigue Severity Scale (FSS), Epworth Sleepiness Scale (ESS), Pittsburgh Sleep Quality Index (PSQI), and the Non-Motor Symptom Assessment Scale (NMSS). Scales were selected to carefully represent the myriad of non-motor symptom domains reflected in PD. Motor assessments were assessed using the Movement Disorders Society-Unified PD Rating Scale (MDS-UPDRS) Parts II (Motor Experiences of Daily Living) and III (Motor Assessment). We also looked at the quality of life (QoL) in participants, utilizing the European Quality of Life scale 5 Dimensions (EQ-5D-3L) questionnaire and the 8-item Parkinson's Disease Questionnaire (PDQ-8) for PD participants. The EQ-5D-3L was selected as an appropriate and widely used measure across various PD population and study cohorts (EuroQol Group, 1990). The EQ-5D scores were converted to a utility index value for Singapore reference population (20) for analysis. Fewer non-motor symptoms and better functioning are indicated by lower scores in non-motor assessments and higher QOL measure scores respectively.

## Statistical Analysis

Statistical analysis was performed using SAS software version 9.4 for Windows (Cary, NC: SAS Institute Inc.). Continuous variables were reported as mean ± standard deviation and Median (interquartile), and categorical variables were reported as frequency (%). PASE, motor and non-motor characteristics were reported at baseline and follow-up by mean ± standard deviation, and the change over 1 year was reported as mean change and 95% confidence interval and tested using paired *t*-test. Median split was conducted to compare the demographical differences of participants with lower and higher physical activity.

Univariate and multivariate regression analyses were performed to investigate the association of physical activity with motor, non-motor and quality of life scores. The estimates of the changes were adjusted for age, gender, ethnicity, years of education, MDS-UPDRS part 3, LEDD, motor subtypes, and baseline measures in the multivariable regression analysis. Change scores from follow-up were calculated by subtracting the baseline scores from the follow-up scores. PASE scores were then regressed to the change scores, adjusting for baseline variables, to find the association of decline or increase in scores (21). Statistical significance was set at *p* < 0.05.

All values were converted to z-scores and regression weights were standardized for ease of interpretation of values. As the study is primarily exploratory in nature without a targeted hypothesis, we did not control for multiple comparisons in the study.

## Results

The cohort studied comprised 121 early PD participants (mean age 64.5 ± 8.2), with a predominantly Han Chinese ethnic group. The baseline characteristics of the study population are presented in Table 1. We divided our participants into two different groups in a median split with higher (>150) and lower (< 150) baseline PASE scores, respectively. PD participants with higher baseline PASE (higher physical activity) had significantly younger age (61.25 ± 7.95 vs. 67.64 ± 7.26, *p* < 0.001), lower motor scores (MDS-UPDRS part 3) (18.56 ± 8.17 vs. 22.80 ± 10.96, *p* = 0.018), lower LEDD (157.43 ± 127.53 vs. 216 ± 153.02, *p* = 0.024), lower memory and attention domain scores on the NMSS (1.00 ± 1.81 vs. 2.62 ± 4.25, *p* = 0.008) and better self-reported QoL (79.6 ± 10.3 vs. 74.7 ± 13.7, *p* = 0.031) as compared to those with lower baseline PASE scores at baseline (Table 1). There were no significant group differences for ethnicity, gender, education or PD subtypes, or any other non-motor symptoms at baseline in relation to PASE. Analyses of PASE sub-scores revealed that participants in the higher PASE group had significantly higher physical activity in the leisure, household and work domains than the low PASE group, with the work sub-score demonstrating the greatest mean difference (81.96 ± 9.00, *p* < 0.001).

**Table 1:** Demographical features of low and high activity groups.

**Variable**	**Whole (*n* = 121)**	**Low PASE (<150) (*n* = 61)**	**High PASE (>150) (*n* = 60)**	***p*-value^+^**
**Demographics**				
Age (year)	64.48 ± 8.23^*^ 65.54 (59.2–69.38)	67.64 ± 7.26 68 (63.91–72.49)	61.25 ± 7.95 62.76 (57.68–67)	** <0.001**
Chinese Ethnicity	106 (87.6)	51 (83.6)	55 (91.7)	0.177
Male Gender	74 (61.16)	36 (59.0)	38 (63.3)	0.628
Education (year)	10.48 ± 4.25 10 (8–13)	10.04 ± 4.41 10 (8–12)	10.92 ± 4.07 10 (8–13)	0.258
**Clinical features**				
MDS-UPDRS Part 3	22.33 ± 8.71 21 (16–28.5)	22.80 ± 10.96 20 (15–29.5)	18.56 ± 8.17 18 (12–25)	**0.018**
PD Subtype				0.528
TD	66 (54.5)	35 (57.4)	31 (51.7)	
PIGD	42 (34.7)	20 (32.8)	22 (36.7)	
Mixed	13 (10.7)	6 (9.8)	7 (11.7)	
LEDD	186.96 ± 143.42 187.5 (60–287.5)	216 ± 153.02 187.5 (100–375)	157.43 ± 127.53 131.5 (50–233.13)	**0.024**
**Assessments**				
MOCA	24.9 ± 3.53	24.6 ± 3.59	25.2 ± 3.47	0.345
HADS-Anxiety	2.62 ± 2.87	2.52 ± 2.73	2.70 ± 2.99	0.739
HADS-Depression	2.98 ± 2.63	3.39 ± 3.04	2.55 ± 2.08	0.078
Apathy Scale	8.74 ± 6.27	9.70 ± 6.37	7.75 ± 6.07	0.087
Fatigue Severity Scale	3.20 ± 1.49	3.32 ± 1.44	3.07 ± 1.54	0.355
Epworth Sleepiness	6.18 ± 3.83	6.33 ± 3.97	6.03 ± 3.73	0.675
PSQI	4.70 ± 3.12	4.59 ± 3.43	4.82 ± 2.79	0.692
**NMSS**
D1: Cardiovascular	0.74± 1.72	0.93 ± 2.05	0.55 ± 1.31	0.222
D2: Sleep and Fatigue	3.02 ± 4.61	2.93 ± 4.03	3.10 ± 5.17	0.844
D3: Mood and Cognition	1.89± 4.31	2.13 ± 5.06	1.65 ± 3.14	0.542
D4: Perceptual Problems and Hallucinations	0.52 ± 1.39	0.49 ± 1.42	0.55 ± 1.37	0.819
D5: Attention and Memory	1.82 ± 3.36	2.62 ± 4.25	1.00 ± 1.81	**0.008**
D6: Gastrointestinal	2.28 ± 3.89	2.54 ± 4.49	2.02 ± 3.31	0.461
D7: Urinary	4.75 ± 5.20	4.90 ± 5.10	4.60 ± 5.34	0.751
D8: Sexual Dysfunction	1.53 ± 4.00	1.49 ± 3.88	1.57 ± 4.14	0.919
D9:Miscellaneous	2.65 ± 4.23	3.28 ± 4.91	2.02 ± 3.31	0.100
EQ5D-VAS	77.18 ± 12.36	74.7 ± 13.7	79.6 ± 10.3	**0.031**
EQ5D- Utility Score	0.925 ± 0.145	0.913 ± 0.175	0.938 ± 0.106	0.337
PDQ-8	8.53 ± 11.42	8.86 ± 11.9	8.20 ± 10.9	0.751
**PASE subscores**
Leisure	50.12 ± 40.31	35.04 ± 23.82	65.21 ± 47.39	<0.001
Household	55.01 ± 36.13	42.75 ± 27.69	67.48 ± 39.52	<0.001
Work	55.81 ± 64.24	15.15 ± 32.27	97.12 ± 62.31	<0.001

* *Mean ± standard deviation [and Median (inter-quartile), where appropriate] and frequency (%) for continuous and categorical variables, respectively*;

+ *Two independent sample t-test for continuous variables and Chi-square test for categorical variables*.

*LEDD, Levodopa Equivalent Daily Dose; MOCA, Montreal Cognitive Assessment; NMSS, Non-Motor Symptom Scale; HADS, Hospital Anxiety and Depression Scale; EQ5D, EuroQoL 5D; VAS, Visual Analog Scale; PSQI, Pittsburgh Sleep Quality Index; PDQ-8, Parkinson's Disease Questionnaire Short-Form. Bold variables are statistically significant values*.

Participants overall declined in physical activity scores on follow-up, as measured by the PASE [MD: −17.83 (95% CI: −32.3, −3.34), *p* = 0.0163]. Participants also performed significantly worse on various measures on follow-up: increased scores for MDS-UPDRS Parts II and III, as well as decreased scores in QOL (EQ-5D Utility Score and PDQ-8) (Table 2). Both the low PASE group and high PASE group had significant increase in UPDRS II and III scores, and poorer QOL scores (EQ-5D Utility Score and PDQ-8) on follow-up. Additionally, the low PASE group had significant change-over-time increase in anxiety scores as compared to the high PASE group.

**Table 2:** Baseline and follow-up variables of PD patients.

**Variable**	**Baseline^†^**	**1-year follow-up^†^**	**Change over time^††^**		**1-year Follow-up^†^ (Low PASE)**	**Change over time (low PASE)^††^**		**1-year Follow-up (High PASE)^†^**	**Change over time (high PASE)^††^**	
PASE	161.2 ± 89.51	143.4 ± 85.34	−17.83 (−32.31,−3.34)	^*^	–	–	–	–	–	–
MDS-UPDRS II	3.71 ± 3.24	4.55 ± 3.80	0.84 (0.24, 1.43)	^**^	4.50 ± 4.50	0.47 (−1.45, 0.50)	NS	4.60 ± 2.96	1.21 (0.53, 1.90)	^**^
MDS-UPDRS III	20.7 ± 9.87	23.97 ± 10.54	3.26 (1.34, 5.19)	^**^	25.45 ± 10.07	2.65 (−5.58, 0.27)	NS	22.45 ± 10.86	3.88 (1.32, 6.43)	^**^
**Cognition**										
MOCA	24.91 ± 3.54	25.06 ± 3.5	0.15 (−0.39, 0.69)	NS	24.77 ± 3.71	0.16 (−1.06, 0.73)	NS	25.35 ± 3.28	0.13 (−0.76, 0.49)	NS
**Non-Motor**										
HADS-Anxiety	2.61 ± 2.87	2.83 ± 3.18	0.21 (−0.19, 0.62)	NS	3.45 ± 3.36	0.93 (0.38, 1.48)	^*^	2.18 ± 2.86	−0.51 (–.043, 1.07)	NS
HADS-Depression	2.98 ± 2.63	3.31 ± 3.16	0.33 (−0.13, 0.79)	NS	3.77 ± 3.32	0.37 (−1.00, 0.25)	NS	2.83 ± 2.94	0.03 (−0.96, 0.39)	NS
PSQI	4.7 ± 3.12	4.82 ± 3.3	0.12 (−0.44, 0.67)	NS	4.77 ± 3.51	0.18 (−0.95, 0.59)	NS	4.86 ± 3.09	0.05(−0.87, 0.77)	NS
Epworth Sleepiness Scale	6.18 ± 3.84	6.08 ± 4.5	−0.12 (−0.96, 0.72)	NS	6.50 ± 4.54	0.18 (−1.43, 1.07)	NS	5.62 ± 4.44	−0.42 (−0.72, 1.57)	NS
Apathy Scale	8.74 ± 6.28	9.49 ± 6.27	0.75 (−0.5, 0.20)	NS	10.70 ± 6.42	1.00 (−2.89, 0.89)	NS	8.25 ± 5.89	0.50 (−2.18, 1.18)	NS
Fatigue Severity Scale	3.2 ± 1.49	3.34 ± 1.64	0.14 (−0.13, 0.41)	NS	3.32 ± 1.45	0.10 (−0.47, 0.26)	NS	3.07 ± 1.54	0.017 (−0.56, 0.21)	NS
**NMSS**										
Domain 1 Cardiovascular	0.74 ± 1.72	0.95 ± 2.11	0.21 (−0.65, 0.23)	NS	1.14 ± 2.21	0.21 (−0.89, 0.47)	NS	0.76 ± 1.99	0.21 (−0.80, 0.36)	NS
Domain 2 Sleep and Fatigue	3.02 ± 4.61	3.85 ± 5.42	0.84 (−1.77, 0.09)	NS	3.52 ± 4.57	0.59 (−1.70, 0.52)	NS	4.20 ± 6.19	1.10 (−2.64, 0.44)	NS
Domain 3 Mood and Cognition	1.89 ± 4.31	2.81 ± 7.77	0.92 (−1.94, 0.08)	NS	2.95 ± 6.56	0.81 (−1.96, 0.32)	NS	2.68 ± 8.89	1.03 (−2.73, 0.67)	NS
Domain 4 Perceptual Problems and Hallucinations	0.52 ± 1.39	0.54 ± 1.66	0.02 (−0.38, 0.33)	NS	0.57 ± 1.47	0.081 (−0.59, 0.43)	NS	0.51 ± 1.85	0.03 (−0.48, 0.54)	NS
Domain 5 Attention and Memory	1.82 ± 3.36	1.56 ± 2.98	−0.25 (−0.38, 0.79)	NS	1.93 ± 3.69	−0.68 (−0.26, 1.63)	NS	1.18 ± 1.99	0.18 (−0.71, 0.34)	NS
Domain 6 Gastrointestinal	2.28 ± 3.89	2.73 ± 3.83	0.45 (−1.11, 0.21)	NS	2.83 ± 3.46	0.29 (−1.29, 0.70)	NS	2.63 ± 4.20	0.61 (−1.51, 0.28)	NS
Domain 7 Urinary	4.75 ± 5.20	5.39 ± 7.26	0.64 (−1.71, 0.42)	NS	5.72 ± 6.75	0.81 (−2.43, 0.79)	NS	5.06 ± 7.78	0.46 (−1.89, 0.96)	NS
Domain 8 Sexual Dysfunction	1.53 ± 4.00	1.30 ± 3.61	−0.22 (−0.62, 1.07)	NS	1.72 ± 4.54	0.22 (−1.56, 1.10)	NS	0.88 ± 2.29	−0.68 (−0.39, 1.75)	NS
Domain 9 Miscellaneous	2.65 ± 4.23	3.29 ± 5.09	0.64 (−1.58, 0.29)	NS	3.01 ± 4.54	−0.26 (−1.27, 1.79)	NS	3.58 ± 5.61	1.56 (0.49, 2.64)	^**^
**QoL**										
EQ5D-VAS	77.18 ± 12.36	76.42 ± 12.89	−0.76 (−3.19, 1.67)	NS	73.86 ± 13.16	−0.91 (−2.56, 4.40)	NS	79.01 ± 12.15	−0.60 (−2.87, 4.07)	NS
EQ5D- Utility Score	0.93 ± 0.15	0.88 ± 0.22	−0.04 (−0.08, −0.01)	^*^	0.84 ± .26	−0.06 (−0.12, 0.00)	^*^	0.91 ± .15	−0.01 (−0.01, 0.05)	NS
PDQ-8	8.53 ± 11.42	12.61 ± 14.35	4.08 (5.99, 2.17)	^***^	13.14 ± 15.09	4.28 (1.61, 6.96)	^**^	12.06 ± 13.67	3.86 (1.04, 6.68)	^**^

† *Mean ± standard deviation*;

†† *Mean (95% confidence interval)*;

+ *Paired t-test; High and Low PASE groups as compared to baseline in Table 1*;

* *Significant at p < 0.05*;

** *Significant at p < 0.01*;

*** *Significant at p < 0.001*.

*NMSS, Non-Motor Symptom Scale; HADS, Hospital Anxiety and Depression Scale; EQ5D, EuroQoL 5D; PSQI, Pittsburgh Sleep Quality Index; PDQ-8, Parkinson's Disease Questionnaire Short-Form; NS, not significant*.

Univariate analysis revealed baseline higher PASE scores to be associated with a decrease in anxiety scores on follow-up [−0.60, 95% CI (−1.00, 0.20), *p* = 0.0035]. After adjusting for potential clinical confounders in the multivariate regression analysis, higher baseline PASE was also associated with decreased anxiety scores [−0.27, 95% CI (−0.52, −0.01), *p* = 0.0373] and apathy scores [−0.33, 95% CI (−0.60, −0.06), *p* = 0.0174] at 1-year follow-up (Table 3). There was no significant association with changes in other non-motor symptoms or the domains of the NMSS.

**Table 3:** Association analysis of baseline PASE scores and follow-up change variables.

**Change in variable scores**	**Univariate**		Multivariate^*^	
	**β (95% CI)**	***P* value**	**β (95% CI)**	***P* value**
MDS-UPDRS II	0.15 (−0.45, 0.75)	0.6218	0.02 (−0.22, 0.26)	0.870
MDS-UPDRS III	0.75 (−1.19, 2.69)	0.4460	−0.13 (−0.36, 0.09)	0.249
**Cognition**
MOCA	0.04 (−0.51, 0.59)	0.8922	−0.037 (−0.26, 0.19)	0.747
**Non-motor symptoms**
HADS-Anxiety	−0.60 (−1.00, −0.20)	**0.0035**	−0.27 (−0.52, −0.01)	**0.0373**
HADS-Depression	0.05 (−0.41, 0.51)	0.8333	0.12 (−0.22, 0.34)	0.3094
Apathy Scale	−0.24 (−1.51, 1.03)	0.7075	−0.33 (−0.60, −0.06)	**0.0174**
Fatigue Severity Scale	0.00 (−0.26, 0.27)	0.9710	−0.03 (−0.26, 0.19)	0.7809
Epworth Sleepiness Scale	−0.06 (−0.91, 0.80)	0.8988	0.06 (−0.21,0.34)	0.6506
PSQI	−0.06 (−0.62, 0.51)	0.8417	0.01 (−0.25,0.27)	0.9284
**NMSS**
Domain 1 Cardiovascular	0.01 (−0.44, 0.46)	0.9485	−0.23 (−0.54,0.08)	0.1426
Domain 2 Sleep and Fatigue	−0.06 (−1.01, 0.89)	0.9007	0.07 (−0.21,0.35)	0.6372
Domain 3 Mood and Cognition	0.31 (−0.71, 1.33)	0.5514	0.27 (−0.01,0.56)	0.0651
Domain 4 Perceptual Problems and Hallucinations	−0.15 (−0.51, 0.21)	0.4206	0.16 (−0.18, 0.49)	0.3572
Domain 5 Attention and Memory	0.26 (−0.29, 0.81)	0.3458	0.20 (−0.11,0.51)	0.1978
Domain 6 Gastrointestinal	0.36 (−0.31, 1.02)	0.2945	0.19 (−0.05,0.42)	0.1246
Domain 7 Urinary	−0.39 (−1.47, 0.68)	0.4715	0.09 (−0.12,0.32)	0.3996
Domain 8 Sexual Dysfunction	−0.55 (−1.41, 0.30)	0.2047	−0.02 (−0.44,0.04)	0.1135
Domain 9 Miscellaneous	0.69 (−0.25, 1.63)	0.1506	0.07 (−0.16, 0.30)	0.5465
**QoL**
EQ5D-VAS	−0.84 (−3.29, 1.61)	0.4974	−0.05 (−0.29, 0.18)	0.6546
EQ5D- Utility Score	0.03 (−0.01, 0.07)	0.1042	0.12 (−0.08,0.34)	0.2435
PDQ-8	−0.11 (−2.06, 1.84)	0.9139	0.07 (−0.24, 0.26)	0.9557

* *Multivariable linear regression model of baseline physical activity as an independent variable in relation to change scores as dependent variables. In the univariate and multivariate models, each row represents one individual model*.

*Standardized estimates are adjusted for age, gender, ethnicity, years of education, MDS-UPDRS Part 3, LEDD, motor subtypes, and baseline outcome measures*.

*NMSS, Non-Motor Symptom Scale; HADS, Hospital Anxiety and Depression Scale; EQ5D, EuroQoL 5D; PSQI, Pittsburgh Sleep Quality Index; PDQ-8, Parkinson's Disease Questionnaire Short-Form. Bold variables are statistically significant values*.

Additionally, we identified 7 participants who moved from a the low PASE group to a high PASE group and 15 participants who moved from the high PASE group to a low PASE group on follow-up. A separate sensitivity analysis after removing these participants showed that the results are consistent with the above with higher baseline PASE significantly associated with decreases in both anxiety [−0.24, 95% CI (−0.45, −0.04), *p* = 0.021] and apathy scores [−0.36, 95%CI (−0.65, −0.07), *p* = 0.016] at 1 year follow-up.

Higher baseline PASE scores were not associated with a significant change in motor nor QoL measures in both the univariate and multivariate models (Table 3).

## Discussion

To the best of our knowledge, this longitudinal cohort is the first to compare baseline self-reported physical activity with changes in a wide spectrum of motor, non-motor and QoL scores amongst early PD patients.

We found that physical activity significantly declined over the course of 1 year, together with increased motor scores and worsened reported QoL. The worsening scores did not discriminate between high or low PASE groups. This has also been found in numerous other cohorts (12) and reflects the challenges faced by PD patients in maintaining their physical activity in the presence of motor progression even over a short span of a year.

We demonstrated that higher baseline PASE scores were associated with a younger age, with lower motor symptom scores, smaller PD medication doses, better attention and memory domain scores as well as better perceived QoL. This group likely represented younger early PD patients who were still physically active and working, and those with milder symptoms who needed less medications. Studies have shown that barriers to overall physical activity are likely similar to that of employment, such as slowness and the fear of falling (22). It is therefore important that PD patients continue to participate in physical activities apart from work, such as household chores, especially upon job retirement (22, 23).

The participants' detailed changes in motor, non- motor, and QoL symptoms were obtained and analyzed at baseline and at 1-year follow-up. These changes were then compared with their baseline physical activity. There was a significant association of physical activity with changes in anxiety and apathy in PD patients longitudinally. Greater baseline physical activity corresponded to a decline in anxiety and apathy in PD on follow-up even though anxiety and apathy scores were not associated with the level of physical activity at baseline. This study suggests that baseline physical activity, even at the early stages of diagnosis, can exert significant benefits in the mood symptoms of PD patients.

Amongst the non-motor measures, early PD participants with greater physical activity experienced improvement in anxiety scores on follow-up. Anxiety is common in PD (31% point prevalence) and can occur in any stage including the pre-motor phase. The clinical correlates of anxiety disorders in PD include a younger age, female gender, worse motor fluctuating scores and past history of neuropsychiatric symptoms (24). Our findings are consistent with a meta-analysis by Wu et al. who reviewed four studies that measured anxiety as a secondary outcome of physical activity and found improvements in anxiety with various exercise interventions (25). However, these studies focused on specific prescribed exercises (i.e., treadmill, aerobic training, qigong, and taichi) over short periods of time (24 weeks−6 months) with no follow-up after the study period (25). A recent longitudinal study also showed that greater physical activity at year 2 of follow-up was associated with slower progression of anxiety (12). The mechanism whereby physical activity and exercise decreases anxiety is not entirely known. Complex interactions of psychological and neurobiological mechanisms are hypothesized (26). The biological effects of increased physical activity and exercise include changes in hypothalamic adrenal axis; and increased secretion of atrial natriuretic peptide, serotonin, amine metabolites and beta endorphins (27).

A decrease in apathy scores on follow-up was also observed in patients with greater baseline physical activity. Apathy in PD has been thought to be a result of reduced limbic and ventral striatal dopamine, and dopaminergic medication may be of some help (28). This is interesting as apathy and depression are often concomitant (29), and present simultaneously. However, studies have also indicated that apathy in PD may also be a separate nosological entity (29, 30). Several cross-sectional studies have also found greater apathy in patients with lower physical activity (31), and to our best knowledge no longitudinal studies on the association of physical activity with apathy have been performed. There is currently no effective treatment for apathy in PD, and apathy can also prevent patients from engaging in treatment, physical and social activities. Studies have found apathy to be crucial factor in the enablement of goal-directed motivation toward physical activity (32). We postulate that a greater baseline physical activity can break the cycle of apathy and reduced self-help behavior that in turn can reduce other symptoms in PD (33).

Further studies are needed to establish the pathophysiological effects of physical activity on anxiety and apathy. While we found that decreased anxiety and apathy were associated with greater baseline physical activity in our study, we are aware of the possibility that participants with greater anxiety and apathy would engage in less physical activity, that in turn could lead to a downward spiral of worsening anxiety and apathy over time. However, we found no significant differences in anxiety and apathy scores between high and low physical activity groups at baseline. Studies have shown that greater dedication and effort are needed by caregivers to motivate interest in physical activity (33, 34). Consistent structured planned physical activities, not limited to exercise, can effectively reduce the reluctance to be active and can prevent the downward spiral of affective conditions in relation to activity in PD (34). We propose that daily physical activity can improve anxiety and apathy in early PD.

Our study had several strengths. This independent longitudinal study cohort was free from physical disabilities which could have contributed to the low physical activity; we also corrected for effects of age, gender, education, medications, motor scores and PD subtyping which could affect or are the major deterrents for physical activity (34). This cohort also underwent a comprehensive evaluation of non-motor, quality of life and motor symptoms. The PASE also enables assessment of an individual's total physical activity that provides a more complete picture of physical activity as compared to measuring exercise or activities that could be performed only episodically. We recognize that self-reported physical activity measures may have potential recall bias. To mitigate this in our study, assessors presented the questions in reference to the period of “over the last week,” as well as to corroborate their monthly history of activities with their caregivers.

In conclusion, our longitudinal study demonstrated that greater baseline physical activity in early PD was associated with decreased anxiety and apathy scores over time, even though baseline anxiety and apathy scores did not differentiate between high and low activity groups. Future studies could incorporate longer follow-up durations and the use of technology devices, such as movement sensors, as well as brain imaging to provide objective measures of physical activity and affective conditions so that we can further decipher the causal relationship between physical activity and mood symptoms.

## Data Availability Statement

The raw data supporting the conclusions of this article will be made available by the authors, without undue reservation.

## Ethics Statement

The studies involving human participants were reviewed and approved by Singapore Health Institutional Review Board. The patients/participants provided their written informed consent to participate in this study.

## Author Contributions

SN and MA: research project execution, statistical analysis, manuscript preparation, and review. ES: statistical analysis. NC, XC, DH, ZX, K-YT, and W-LA: research project execution. E-KT: research project design and execution. LT: research project design, manuscript preparation, and review. All authors contributed to the article and approved the submitted version.

## Conflict of Interest

The authors declare that the research was conducted in the absence of any commercial or financial relationships that could be construed as a potential conflict of interest.
